# Heliox in the treatment of status asthmaticus: case reports

**DOI:** 10.5935/0103-507X.20160005

**Published:** 2016

**Authors:** Inês Carvalho, Sara Querido, Joana Silvestre, Pedro Póvoa

**Affiliations:** 1 Polyvalent Intensive Care Unit, Hospital de São Francisco Xavier, Centro Hospitalar de Lisboa Ocidental - Lisboa, Portugal.; 2 Center of Studies on Chronic Diseases, Faculdade de Ciências Médicas, Universidade Nova de Lisboa - Lisboa, Portugal.

**Keywords:** Heliox, Airway obstruction, Asthma, Acute disease, Respiration, artificial, Case reports

## Abstract

Helium was discovered in 1868 by the French astronomer Pierre-Jules-César Janssen and was first used as a therapeutic treatment for airway obstruction by Barach almost 70 years later, in 1934. Heliox is characterized by its low density, which makes it more fluid under conditions of turbulence, thus minimizing airway pressure and facilitating the occurrence of laminar flow. The present article describes two clinical cases of patients with status asthmaticus subjected to mechanical ventilation and refractory to treatment in whom heliox was used, which allowed optimization of the efficacy of conventional pharmacological treatments. Although heliox is still used sporadically and its true efficacy has not been well demonstrated, the unique physical properties of helium and the theoretical improvement of the airflow in obstructed airways have produced scientific interest and stimulated research. Heliox can be used simultaneously with conventional therapies in cases of serious and refractory exacerbations of severe obstructive disease.

## Abstract (pt)

O hélio foi descoberto em 1868 pelo astrônomo francês Pierre-Jules-César Janssen e teve seu uso terapêutico pela primeira vez na obstrução das vias aéreas, feito por Barach, quase 70 anos depois, em 1934. O heliox é caracterizado por sua baixa densidade, o que lhe confere melhor fluidez sob condições de turbulência, minimizando a pressão das vias aéreas e facilitando a ocorrência de um fluxo laminar. Este artigo apresenta dois casos clínicos de doentes com mal asmático sob ventilação mecânica, refratários à terapêutica, em que se recorreu ao heliox, permitindo uma otimização da eficácia do tratamento farmacológico convencional. Apesar de sua utilização permanecer esporádica e sua verdadeira eficácia não se encontrar bem demonstrada, as propriedades físicas únicas do hélio e a melhoria teórica do fluxo de ar nas vias aéreas obstruídas fomentam o interesse e a pesquisa científicos. Sua aplicação pode ter lugar simultaneamente em terapêuticas convencionais nas exacerbações graves e refratárias da doença obstrutiva grave.

## INTRODUCTION

Helium (He) was discovered in 1868 by the French astronomer Pierre-Jules-César Janssen.^(1,2)^ In 1934, Barach described the first application of He for the treatment of airway obstruction and asthma exacerbations and demonstrated that its low density reduced the work of breathing and improved ventilation.^(3-5)^

Since that time, several mixtures of He and oxygen (so-called "heliox" - He/O_2_) have been used for the treatment of several respiratory problems, such as exacerbations of asthma and chronic bronchitis, i.e., obstructive diseases associated with increased expiratory resistance.^(1,2,5)^

He/O_2_ can be used with any oxygen delivery device, including nasal cannulas, face masks, noninvasive ventilation, and conventional mechanical ventilation devices.^(1,4,6)^

**Figure 1:**
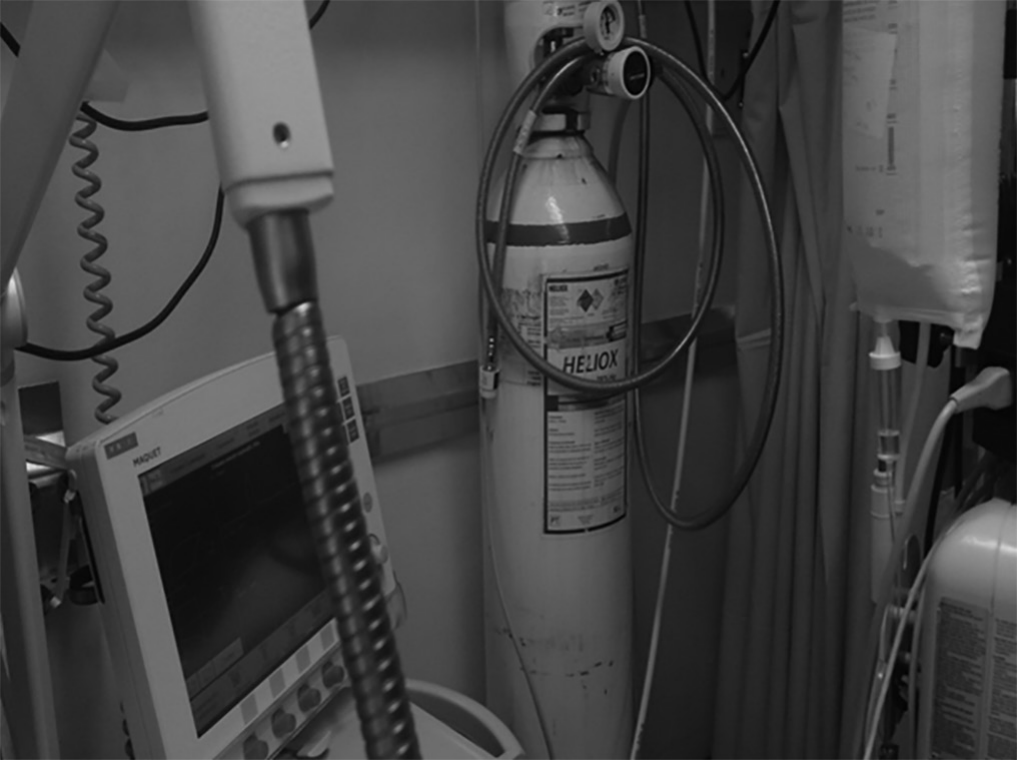
Use of heliox (80:20) with a Maquet Servo-I ventilator.

Some requirements are needed for heliox to be used during invasive mechanical ventilation, including the availability of a ventilator (e.g., the Maquet Servo-i) with a specific module for automatic recalibration of the replacement of air by He/O_2_ (supplied by pressurized bottles in a ratio of 80% He:20% O_2_ - Figure 1). The fraction of inspired oxygen (FiO_2_) is titrated as a function of the oximetry target values. The effect of He/O_2_ on the ventilatory mechanics is inversely proportional to the FiO_2_ in the inspired gas mixture.

Except for the Maquet Servo-i, volume readings are inaccurate on all ventilators because the flow meter is calibrated for air.^(1)^ Furthermore, the dosing of the amount of administered aerosols is also affected by the type of gas used. It should be noted that no adverse effects associated with the use of He/O_2_ have been described.^(4,7)^ Therefore, administration of He/O_2_ is safe and does not require the use of any type of gas exhaustion system.

### Literature review

From the 1930s to the present time, several studies have evaluated the impact of He/O_2_ delivery on the maximum flow rate, airway peak pressure, dyspnea scores, and performance in pulmonary function tests. However, the data provided by such studies were often contradictory concerning the usefulness of He/O_2_ in pulmonary obstructive disease.^(1,3)^

Colebourn et al *.*^(8)^ conducted a systematic review of all of the controlled randomized trials that compared He/O_2_ to air-oxygen mixtures in patients with acute exacerbations of asthma and chronic obstructive pulmonary disease. The primary outcome measures in the studies of asthma included changes in spirometric parameters, namely forced expiratory volume in one second, forced vital capacity, and forced expiratory flow; the secondary outcomes consisted of clinical (dyspnea score and respiratory rate) and analytical (arterial oxygen saturation) parameters and hospital admission rate. The results did not indicate benefits of the use of He/O_2_ in routine treatment; however, the studies were small and exhibited methodological flaws.

Rodrigo et al.^(7)^ performed a systematic review with a meta-analysis of randomized clinical trials comparing the efficacy of He/O_2_ and oxygen for driving beta2 agonist nebulization in patients with acute asthma. The primary outcomes were change in spirometric parameters and severity scores; the secondary outcomes were hospital admission rate and severe adverse effects. The patients who received He/O_2_ presented a statistically significant improvement of the peak expiratory flow of 20L/m (95% confidence interval - 95%CI: 5.2 - 29.4; p = 0.005), which was more evident in the subgroup of patients with severe and very severe asthma).

He/O_2_ also produced significant decreases in the hospital admission rate (odds ratio - OR 0.49; 95% CI: 0.31 - 0.79; p = 0.003). The groups did not differ in the occurrence of serious adverse effects. Therefore, it may be concluded that the benefits of He/O_2_ on airflow limitation associated with airway obstruction as well as on hospital admission rate are clinically significant.

Few reports in the literature describe the use of He/O_2_ in patients subjected to mechanical ventilation, and most consist of retrospective studies and case reports; no controlled studies have been conducted with intubated patients.^(1)^

**Figure 2:**
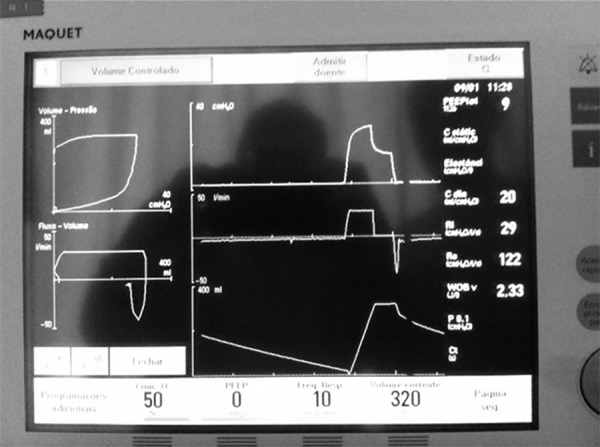
Parameters and ventilatory mechanics before heliox administration. Controlled hypoventilation protocol.

Our ventilation protocol for patients with severe obstructive disease was based on controlled hypoventilation; volume-controlled ventilation with high inspiratory output (approximately 60L/min) was combined with controlled hypoventilation (low respiratory rate: 8 - 10 cycles per minute (cpm) when the patient tolerated a pH of 7.25-7.28), a positive end-expiratory pressure (PEEP) of 0 to optimize the expiratory time, and limitation of the plateau pressure (Pplateau) to 30cmH_2_O (Figure 2).

As the present study was a case report, informed consent was waived by the local ethics committee.

## CASE REPORTS

### Clinical case 1

The case of a male, 39-year-old patient with history of bronchial asthma since childhood, irregular treatment (budesonide and terbutaline), and without outpatient monitoring is described. The patient had an episode of status asthmaticus requiring invasive mechanical ventilation approximately 10 years earlier.

The patient was brought to the emergency department due to dyspnea and wheezing that began 24 hours earlier and did not improve after short-acting bronchodilator therapy with salbutamol. Upon admission, the patient was in state of respiratory exhaustion; he was subjected to orotracheal intubation and connected to a ventilator, and bronchodilator therapy was started. Clinical, laboratory, and radiological assessments did not detect evidence of infection.

Due to the need for ventilatory support, the patient was transferred to the intensive care unit (ICU), where bronchodilator therapy and sedoanalgesia were optimized with administration of midazolam, alfentanil, and ketamine. Curarization was performed with vecuronium, and a controlled hypoventilation protocol was started. In terms of ventilatory mechanics, the patient exhibited increased expiratory time, severe bronchospasm, and a marked expiratory flow limitation pattern (time constant (τ), 1.69 seconds). In addition to the hypoventilation strategy, continuous high-dose inhaled and intravenous corticosteroid therapy (methylprednisolone) and magnesium sulfate were added to the nebulized bronchodilator therapy. Because his IgE levels were high, omalizumab was also administered.

On the 9^th^ day of admission, the patient still exhibited global respiratory failure, with severe acidemia and persistent expiratory flow limitation (peak inspiratory pressure (Ppeak), 57cmH_2_O; Pplateau, 25cmH_2_O; static compliance (Cs), 45mL/cmH_2_O; τ, 3.55 seconds; intrinsic PEEP (PEEPi), 1).

Ventilation with He/O_2_ was started (and maintained for three days), which produced significant improvement of the airway resistance and obstructive pattern, as shown by reduction of the peak pressure, resistance, τ, and PEEPi (Table 1).

**Table 1:** Ventilation, blood gases, and ventilatory dynamics parameters during the hospital stay

**Admission day**	**Clinical case 1**									**Clinical case 2**								
	**Pre-heliox**			**Heliox**			**Post-heliox**			**Pre-heliox**			**Heliox**			**Post-heliox**		
	**1**	**2**	**6**	**7**	**9**	**11**	**12**	**14**	**19**	**1**	**2**	**5**	**6**	**9**	**10**	**13**	**19**	**20**
Ventilator parameters																		
Modality	VC	VC	VC	VC	VC	VC	PS	SV	SV	VC	VC	PC	VC	PC	PS	SV	SV	SV
FiO_2_	100	55	60	60	40	50	50	60	40	40	45	65	30	50	40	60	28	28
Frequency	12	10	10	10	10	12				15	12	14	12	16				
Inspiratory pressure							10					19		20	10			
PEEPe	0	0	0	0	0	0	4			5	5	0	0	0	4			
Tidal volume	300	370	480	480	410	500				420	400		370					
I:E ratio	1:4	1:6.7	1:6.7	1:6.7	1:6.7	1:5.0				1:2.0	1:2.0	1:4.0	1:5.0	1:3.0				
Ventilatory mechanics																		
Pplateau	23	22	13	19	25	10				21	20	25	20	15				
Ppeak		58	58	60	57	28				46	44	50	48	24				
Cs	21	21	28	37	45	50				28	31	27	31	45				
Resistance		80	68	82	80	24				43	60	155	140	30				
τ	3.79	1.69	1.9	3.03	3.55	1.2				1.19	1.86	4.18	4.34	1.34	0.6			
PEEPi	9	4	3	6	1	0				1	1	5	5	1	0			
Arterial blood gases																		
pH	7.29	7.08	7.37	7.22	7.34	7.44	7.48	7.46	7.47	7.3	7.43	7.43	7.35	7.44	7.48	7.51	7.46	7.45
PaCO_2_	53.9	112	73.5	108	77.1	49.4	32.1	33.2	32.9	47	38.5	48.5	58.9	52	43.5	36.7	44.7	47.6
PaO_2_	493	104	112	125	139	126	76.2	112	107	165	100	273	97.7	80	144	87.7	135	148
HCO_3_	23.1	11.2	37.6	35.4	36.1	29.1	25.2	24.6	24.6	21.5	24.6	24.5	29.5	33.9	32	30.4	31.2	33.4
BE	-0.2	2.2	15.3	14.8	14.3	5.7	0.3	-0.2	-0.2	-3	0.1	0.6	6.5	10	8	6.3	7.6	9
SaO_2_	99.1	97.1	98.5	98.1	96.6	98.6	96.3	97.5	97.7	99.1	98	99.2	98.1	97	98.9	98.5	99.4	99.4
Lactate	4	0.5	1.3	0.4	0.4	1.3	1.5	0.7	0.8	2.3	3.3	1.1	1.3	0.7	1.3	3.3	1.4	1.1

VC - volume-controlled ventilation; PS - pressure support ventilation; SV - spontaneous ventilation; PC - pressure-controlled ventilation; FiO_2_ - fraction of inspired oxygen; PEEPe - extrinsic positive end-expiratory pressure; I:E - inspiration:expiration; Pplateau - plateau pressure; Ppeak - peak inspiratory pressure; Cs - static compliance; τ - time constant; PEEPi - intrinsic positive end-expiratory pressure; PaCO_2_ - partial pressure of carbon dioxide; PaO_2_ - partial pressure of oxygen; HCO_3_ - bicarbonate; BE - base excess; SaO_2_ - arterial oxygen saturation.

On the 11^th^ day, progressive and consistent improvement of the blood gases was observed, curarization was discontinued, and sedation was decreased. On the 16^th^ day, the patient was weaned from the ventilator and extubated without any complications. The patient was free from hospital-acquired infections during his stay at the hospital, and thus, no antibiotic therapy was required.

The patient exhibited gradual clinical improvement without fever, was stable with regard to ventilation and hemodynamic parameters, and maintained adequate blood gases with an oxygen supply of 5L/min via a nasal cannula (Table 1).

On the 19^th^ day, because he no longer required intensive care, the patient was transferred to a hospital near his place of residence.

Hospital discharge occurred on the 30^th^ day.

### Clinical case 2

The case of a female, 53-year-old patient with a history of bronchial asthma, followed-up at a pneumology service since the age of 30, is described. The patient had several hospital admissions for decompensated asthma (requiring ventilatory support on two occasions) and one episode of seizures related to decompensated asthma. She was regularly treated with budesonide, formoterol, montelukast, omeprazole, hydroxyzine, and home oxygen therapy (2L/min).

The patient was transported to the emergency department for dyspnea starting 36 hours earlier and progressed into respiratory arrest, resulting in orotracheal intubation and mechanical ventilation.

Due to her state of status asthmaticus/respiratory failure, she was transferred to the ICU, where her condition improved during the first 48 hours and was stable with regard to hemodynamic and ventilation parameters. Extubation was attempted, but it was complicated by immediate occurrence of severe bronchospasm.

On the 5^th^ day, the patient exhibited an episode of severe hypoxia, requiring an increase in sedation, curarization, and adjustment of bronchodilators. Regarding her ventilatory parameters, she exhibited marked expiratory flow limitation (Cs, 27mL/cmH_2_O; τ, 4.18 seconds; PEEPi, 5) due to severe bronchospasm.

The controlled hypoventilation protocol was restarted and combined with He/O_2_ (for four days) and inhaled intravenous corticosteroid therapy using methylprednisolone and montelukast (10mg/day).

The parameters of ventilatory dynamics exhibited significant improvement, namely reduction of the Pplateau, resistance, τ, and PEEPi (Table 1).

Weaning from mechanical ventilation was complicated by episodes of bronchospasm; eventually she was extubated on the 18^th^ day. The patient was free from hospital-acquired infections and was not subjected to antibiotic therapy.

At the time of transfer (20^th^ day), the patient was awake, calm, and cooperative, with efficient cough and preserved swallowing reflex, in addition to liquid respiratory secretions. The blood gases were adequate, with an oxygen supply of 2L/min through a nasal cannula, and she was capable of standing in a chair.

Hospital discharge occurred on the 40^th^ day.

## DISCUSSION

Many of the recently published studies on the use of He/O_2_ for refractory asthma have concluded that such treatments aim to reduce airflow resistance in obstructed airways, with consequent improvement of alveolar ventilation and of the efficacy of bronchodilator therapy by increasing the diffusion of drugs so that they might reach the smaller airways.^(5,7,9,10)^

Currently, He/O_2_ has been mainly used in both pediatric and adult patients as adjuvant treatment while waiting for the onset of effects of conventional pharmacological treatments.^(5,6)^ In the two clinical cases described here, we observed improvement of airway resistance to flow as well as the alveolar ventilation (demonstrated in the tables by progressive reduction of Ppeak, resistance, τ, and PEEPi). Thus, we believe that He/O_2_ might represent an option for refractory upper airway obstruction treatment in patients exhibiting progressive aggravation despite optimized ventilatory strategies and pharmacological treatment.

The use of He/O_2_ in mechanically ventilated patients has only been described in the literature in retrospective studies and case reports; no controlled studies of intubated patients have been reported to date.

Thus, recognizing the limitations of the present study, we consider the performance of controlled randomized studies essential to assess the identified benefits of He/O_2_ in adult patients subjected to invasive mechanical ventilation.

## CONCLUSION

Heliox is still sporadically used, and its true efficacy has not been well demonstrated. It appears to be an appropriate gas for oxygen and pharmacological treatment delivery, in respiratory diseases, due to its characteristic low density. It can be used simultaneously with conventional treatment for serious and refractory exacerbations of severe obstructive disease.

The level of evidence currently available does not allow a formal recommendation of heliox use in the intensive care unit; however, further clinical studies are required.
